# Genetic diversity of a New Zealand multi-breed sheep population and composite breeds’ history revealed by a high-density SNP chip

**DOI:** 10.1186/s12863-017-0492-8

**Published:** 2017-03-14

**Authors:** Luiz F. Brito, John C. McEwan, Stephen P. Miller, Natalie K. Pickering, Wendy E. Bain, Ken G. Dodds, Flávio S. Schenkel, Shannon M. Clarke

**Affiliations:** 1 Centre for Genetic Improvement of Livestock, University of Guelph, Guelph, N1G 2W1 Canada; 2 AgResearch, Invermay Agricultural Centre, Private Bag 50034, Mosgiel, 9053 New Zealand; 3 Focus Genetics, Napier, 4110 New Zealand

**Keywords:** Crossbreeding, Effective population size, Linkage disequilibrium, Consistency of gametic phase, Population structure, Sheep

## Abstract

**Background:**

Knowledge about the genetic diversity of a population is a crucial parameter for the implementation of successful genomic selection and conservation of genetic resources. The aim of this research was to establish the scientific basis for the implementation of genomic selection in a composite Terminal sheep breeding scheme by providing consolidated linkage disequilibrium (LD) measures across SNP markers, estimating consistency of gametic phase between breed-groups, and assessing genetic diversity measures, such as effective population size (N_e_), and population structure parameters, using a large number of animals (*n* = 14,845) genotyped with a high density SNP chip (606,006 markers). Information generated in this research will be useful for optimizing molecular breeding values predictions and managing the available genetic resources.

**Results:**

Overall, as expected, levels of pairwise LD decreased with increasing distance between SNP pairs. The mean LD r^2^ between adjacent SNP was 0.26 ± 0.10. The most recent effective population size for all animals (687) and separately per breed-groups: Primera (974), Lamb Supreme (380), Texel (227) and Dual-Purpose (125) was quite variable. The genotyped animals were outbred or had an average low level of inbreeding. Consistency of gametic phase was higher than 0.94 for all breed pairs at the average distance between SNP on the chip (~4.74 kb). Moreover, there was not a clear separation between the breed-groups based on principal component analysis, suggesting that a mixed-breed training population for calculation of molecular breeding values would be beneficial.

**Conclusions:**

This study reports, for the first time, estimates of linkage disequilibrium, genetic diversity and population structure parameters from a genome-wide perspective in New Zealand Terminal Sire composite sheep breeds. The levels of linkage disequilibrium indicate that genomic selection could be implemented with the high density SNP panel. The moderate to high consistency of gametic phase between breed-groups and overlapping population structure support the pooling of the animals in a mixed training population for genomic predictions. In addition, the moderate to high N_e_ highlights the need to genotype and phenotype a large training population in order to capture most of the haplotype diversity and increase accuracies of genomic predictions. The results reported herein are a first step toward understanding the genomic architecture of a Terminal Sire composite sheep population and for the optimal implementation of genomic selection and genome-wide association studies in this sheep population.

**Electronic supplementary material:**

The online version of this article (doi:10.1186/s12863-017-0492-8) contains supplementary material, which is available to authorized users.

## Background

Sheep farming is of significant economic importance to New Zealand and is represented throughout the country. The variable climates and landscapes have favoured the adoption of a wide diversity of sheep breeds that have adapted and performed well for different breeding objectives (Maternal vs Terminal) under a range of production systems (e.g. intensive vs extensive). Although there are a significant number of purebred sheep farms, over time the New Zealand sheep industry has been characterized by a high and increasing proportion of composite breeds and crossbreed animals [1, 2]. As described by Blair [1], New Zealand sheep farmers are largely focused on profitability of their stock compared to that of raising solely purebred animals.

Genomic selection (GS) [3] has played an important role on increasing profitability in livestock species by improving selection efficiency. The success of GS depends on many factors such as the extent of the Linkage Disequilibrium (LD, the non-random association of alleles at different loci) across the genome, which may vary between breeds/populations. The history of the population under selection and its genetic diversity has implications on the long-term success of a breeding program (genetic gains per generation that can be achieved) and determines cost effective tools/ways to apply GS (e.g. SNP chip density) [4]. Over the last 30 years several composite breeds have been developed in New Zealand for a commercial need, however their genetic diversity is still unknown and their breeding history has not been fully documented in the scientific literature. Some of these composite breeds are Primera and Lamb Supreme. Therefore, to enable GS and characterise the genetic diversity in the New Zealand Terminal Sire composite breeds, a high density SNP array (606,006 SNPs) was commissioned by FarmIQ™ (joint New Zealand government and industry Primary Growth Partnership) and developed in conjunction with the International Sheep Genomics Consortium (ISGC) and Illumina [5, 6].

The main objectives of this study were: 1) to collate and present the breeding history of new composite breeds widely raised in New Zealand and overseas; and 2) to establish the scientific basis for the implementation of genomic selection in a composite Terminal breeding scheme by: providing consolidated LD measures across SNP markers; estimating consistency of gametic phase between breed-groups; and, estimating other genetic diversity measures relevant for the successful predictions of molecular breeding values (mBVs), such as N_e_, pedigree and genomic inbreeding, and population structure. This investigation will also provide fundamental information related to the genomic architecture of this sheep population.

## Methods

### Genotype data and quality control

There were 14,845 animals from both sexes (7,961 males and 6,884 females) with HD (Ovine Infinium® HD SNP Beadchip) genotype call rate greater than 95%. The animals were born in: 2007–2009 (*n* = 208); 2010 (*n* = 3,623); 2011 (*n* = 3,782), 2012 (*n* = 2,383), 2013 (*n* = 2,175) and 2014 (*n* = 2,674). DNA was extracted mostly from ear punch tissue [7]; however, DNA was also extracted from blood [8] and semen samples as well. Genotyping was conducted at the AgResearch Animal Genomics Research Laboratory, Mosgiel, New Zealand.

Genotypes were called on the AB system and using Illumina GenomeStudio® software. Genotypes were coded as the number of A alleles (0, 1 or 2). SNP were excluded from the analysis if their minor allele frequency (MAF) was less than 0.01, had call rate less than 95%, were non-autosomal, had unknown genomic position on the sheep reference genome assembly version OARV3.1, had duplicated map positions (two SNP with the same position, but with different names), had misplaced SNP positions compared to OARv3.1, and/or showed an extreme departure from Hardy Weinberg equilibrium (*p* < 10^−15^). A total of 517,902 SNP were retained for further analyses after filtering. Following quality control, missing genotypes were minimal (2.16%) and were subsequently imputed using the FImpute software [9]. The analysis were performed for each breed group separately (Primera, Lamb Supreme, Texel, or Dual-Purpose) and using the whole dataset of genotyped animals.

#### Extent of linkage disequilibrium

The degree of LD between markers was estimated using the squared correlation coefficient (r^2^) statistic as proposed by Hill and Robertson [10], which is the squared correlation between alleles at two loci. It can be expressed as: ${r}^2=\frac{D^2}{f\left({A}_i\right) f\left({B}_i\right) f\left({A}_j\right) f\left({B}_j\right)}$, where *f*(*A*_*i*_), *f*(*B*_*i*_), *f*(*A*_*j*_), and *f*(*B*_*j*_), are observed frequencies of alleles A_i_, B_i_, A_j_, and B_j_, respectively and *i* and *j* are markers. D was estimated as suggested by Lynch and Walsh [11]: $D=\frac{N}{N-1}\left[\frac{4{N}_{AABB}+2\left({N}_{AABb}+{N}_{AaBB}\right)+{N}_{AaBb}}{2 N}-2\times f(A)\times f(B)\right],$ where *N* is the total number of animals, and *N*_*AABB*_, *N*_*AABb*_, *N*_*AaBB*_, and *N*_*AaBb*_ are the corresponding number of individuals in each genotypic category (AABB, AABb, AaBB, and AaBb). Considering the r^2^ between a bi-allelic marker and an (unobserved) bi-allelic quantitative trait loci (QTL), r^2^ is the proportion of variation caused by the alleles at a QTL that is explained by the markers [12] and it ranges from 0 (no LD) to 1 (complete LD) between two markers. The r^2^ for each pair of loci on each chromosome was calculated to determine the LD between adjacent and syntenic SNP pairs. LD (r^2^) decay over different distances was also investigated.

#### Consistency of gametic phase

The consistency of gametic phase was defined by the Pearson correlation of signed r-values between two breed-group pairs. For each markers pair with a measure of r^2^, the signed r-value was determined by taking the square root of the r^2^ value and assigning the appropriate sign based on the calculated disequilibrium (D) value. Data was sorted into bins based on pairwise marker distance to determine the breakdown in the consistency of gametic phase across distances. For each distance bin, the signed r-values were then correlated between all six breed-group pairs. The analysis were performed on snp1101 software [13].

#### Current and ancestral effective population size

To estimate N_e_ through time, the formula used was *Ne = ((1/E[r* ^*2*^ ]) – 1)*(1/4c) [14], where *c* is the average genetic distance in Morgans estimated for each chromosome in the LD analysis (estimated using snp1101 package) and *E[r* ^*2*^ *]* is the expected r^2^ at distance *c* calculated as $E\left({r}^2\right)=\frac{1}{1+4{N}_e c}$. Time is in generations, assuming *T = 1/2c* [15]. N_e_ was determined from current to 1,000 generations ago.

#### Principal component analysis

To investigate the genomic composition of the population, the principal components were derived from the genomic relationship matrix (**G**) calculated using all the genotyped animals and all SNPs that passed the quality control process. The **G** matrix was calculated using the method described by VanRaden [16]: $G=\frac{\left( M-2 P\right)\left( M-2 P\right)\boldsymbol{\hbox{'}}}{2\sum {p}_i\left(1-{p}_i\right)}$, where **M** is a matrix of counts of the alleles “A” (with dimensions equal to the number of animals by number of SNP), *p*_*i*_ is the frequency of allele “A” of the i^th^ SNP, and **P** is a matrix (with dimensions equal to the number of animals by number of SNP) with each row containing the *p*_*i*_ values. Principal components were calculated using the *prcomp* function of R [17].

#### Pedigree and genomic inbreeding coefficients

Both pedigree (F_PED_) and genomic inbreeding coefficients in this population were estimated and compared. Pedigree information was available from 243,486 individuals born from 1990 to 2014 and F_PED_ was calculated using the Meuwissen and Luo [18] algorithm. Genomic inbreeding was calculated as:

1. **Inbreeding coefficient based on excess of homozygosity (PLINK software** [19]**, F**_**EH**_ **):** $\frac{1}{m}{\displaystyle {\sum}_{i=1}^m}\kern0.5em 1-\frac{c_i\left(2-{c}_i\right)}{p_i\left(1-0.5{p}_i\right)}$, where *m* is the number of SNP, *p*_*i*_ is the minor allele frequency at loci *i* and *c*_*i*_ is the genotype call (0, 1 or 2).
2. **Diagonal of VanRaden’ G-matrix minus 1 (F**_**VR**_ **):** Genomic relationship matrix was calculated as in VanRaden [16] and the F_VR_ was calculated as the diagonal element minus 1 for each individual.

## Results

### Genotypes

**Fig. 1:**
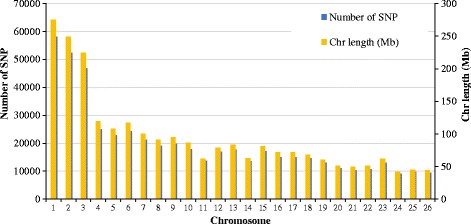
Marker density over the genome represented by the number of SNP (blue bars) and length of chromosome spanned (yellow bars)

**Fig. 2:**
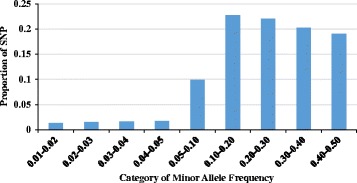
Minor allele frequency distributions for the whole genome after quality control

**Fig. 3:**
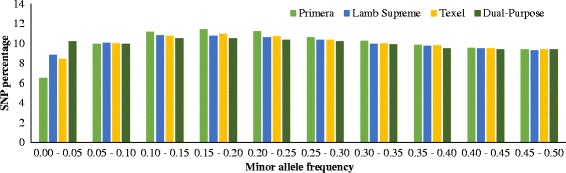
Distribution of SNPs by MAF ranges and breed group

The 517,902 SNP markers that passed quality control spanned about 2.45 Gb of the genome, with an average distance of 4.74 kb between adjacent SNPs, which varied between chromosomes (ranging from 4.50 kb in OAR11 to 4.84 kb in OAR10). Figure 1 presents the number of SNP per chromosome and chromosome length, indicating that SNPs were uniformly distributed across the genome. The number of SNP per chromosome ranged from 58,074 (OAR1, longest chromosome; 42.01 Mb) to 9,191 (OAR24, shortest chromosome; 27.56 Mb). The maximum gaps between adjacent SNPs were observed on OAR5 (305.58 kb), OAR10 (357.01 kb) and OAR13 (343.36 kb). The distribution of MAF of the SNPs after quality control is given in Fig. 2 and the MAF distribution per breed group is shown in Fig. 3. The mean MAF (± SD) over all genotyped animals was 0.255 ± 0.136 and for the breed-groups Primera, Lamb Supreme, Texel and Dual-Purpose was 0.254 ± 0.137, 0.248 ± 0.141, 0.249 ± 0.140 and 0.245 ± 0.143, respectively. SNPs were found to have a broad range of MAF (Fig. 2). The distribution of the MAF shows that the proportion of SNPs with high polymorphism (MAF > 0.3) after quality control was 39.27%. The mean expected heterozygosity (H_e_) for all the genotyped animals was 0.346 (±0.009) and ranged from 0.249 to 0.383. H_e_ (± SD) was 0.350 (±0.006), 0.346 (±0.011), 0.340 (±0.007) and 0.332 (±0.010) for Primera, Texel, Lamb Supreme and Dual-Purpose, respectively.

### Genetic resources

The sheep population under investigation is predominantly focused on breeding for faster growth, higher carcass yield, survival and improved meat quality. The majority of the genotyped animals were progeny of Terminal Sire composites and Texel mated to a variety of maternal/dual-purpose breeds. The main breeds involved were Lamb Supreme, Primera, Texel, Romney, Coopworth, Landmark and Highlander. Due to the lack of literature for some of the composite breeds, we collate a brief history of them, presented in Additional file 1.

### Genomic and pedigree inbreeding

Pedigree (F_PED_) and two genomic (F_EH_, F_VR_) inbreeding coefficients by year of birth were calculated (Table 1). Pedigree inbreeding had the highest average values of the three inbreeding coefficient measures. The average F_PED_ was 0.002 ± 0.009 and ranged from 0.000 to 0.277. The average F_PED_ for the sires was 0.014 and 0.012 for the dams. The average F_PED_ for the inbred animals (F_PED_ > 0) was 0.029. The genomic inbreeding coefficients based on excess of homozygosity (F_EH_) or **G** matrix (F_VR_) were −0.008 ± 0.031 (range: −0.079 – 0.301) and −0.009 ± 0.027 (range: −0.093 – 0.328), respectively. Correlation between F_PED_ and genomic inbreeding was 0.27 (F_EH_) and 0.36 (F_VR_). The correlation between F_EH_ and F_VR_ was 0.51. There were individuals with high genomic inbreeding, but zero pedigree inbreeding (incomplete pedigree information). This highlights another advantage of genomic information for breeding programs.

**Table 1:** Mean inbreeding coefficients (± SD) and inbreeding range per year

	F_PED_		F_EH_		F_VR_	
Birth year	Mean ± SD	Range	Mean ± SD	Range	Mean ± SD	Range
2010	0.0005 ± 0.0049	0.0000 – 0.0744	−0.0165 ± 0.0256	−0.0707 – 0.1270	−0.0145 ± 0.0164	−0.0651 – 0.20137
2011	0.0008 ± 0.0062	0.0000 – 0.1672	−0.0113 ± 0.0290	−0.0790 – 0.3006	−0.0167 ± 0.0214	−0.0933 – 0.3278
2012	0.0017 ± 0.0083	0.0000 – 0.0851	−0.0078 ± 0.0309	−0.0734 – 0.1381	−0.0138 ± 0.0226	−0.0895 – 0.1631
2013	0.0041 ± 0.0128	0.0000 – 0.1569	−0.0030 ± 0.0353	−0.0693 – 0.1825	0.0004 ± 0.0332	−0.0670 – 0.2394
2014	0.0030 ± 0.0118	0.0000 – 0.2776	−0.0047 ± 0.0312	−0.0633 – 0.2675	−0.0003 ± 0.0317	−0.0570 – 0.2806
All	0.0021 ± 0.0095	0.0000 – 0.2776	−0.0087 ± 0.0314	−0.0790 – 0.3006	−0.0091 ± 0.0276	−0.0933 – 0.3278

*F*_*PED*_ pedigree inbreeding coefficient, *F*_*EH*_ inbreeding coefficient based on excess of homozygosity, *F*_*VR*_ inbreeding coefficient based on G matrix (VanRaden), *SD* standard deviation

### Extent of linkage disequilibrium

**Fig. 4:**
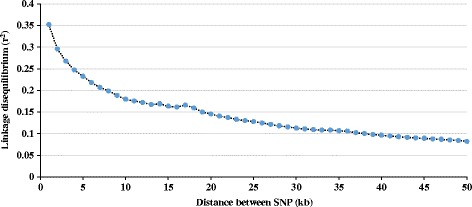
Average linkage disequilibrium (r^2^) at given distances for all animals included in this study

The results of descriptive analysis of SNP markers and LD (r^2^) between adjacent markers obtained for each chromosome are shown in Table 2. The mean r^2^ between adjacent SNPs was 0.263 ± 0.10 and chromosomal mean ranged from 0.244 (OAR26) to 0.282 (OAR13). The LD levels between adjacent markers were also evaluated by breed-group and are presented in Additional file 2. Results from this study reveal some LD variability between the different breed-groups. Dual-Purpose presented the highest LD level (0.274), followed by Lamb Supreme (0.266), Texel (0.261) and finally Primera (0.256). Pairwise r^2^-values were also averaged over all autosomes and plotted as a function of genomic distance between markers (Fig. 4). At the average marker spacing in the HD SNP chip (~5 kb) the average LD (r^2^) was 0.24. Overall, levels of pairwise LD decreased with increasing distance between SNP. For distances between SNPs greater than 8 kb, the LD levels were less than 0.20 and decreased constantly, with exception of two points (up to 14 and 17 kb) where there was a small increase in LD. For SNP located more than 40 kb apart, the LD levels were less than 0.10.

**Table 2:** Average linkage disequilibrium (r^2^) between adjacent SNP pairs by chromosome and including all genotyped animals (*n* = 14,845)

Chr.	N pairs	Mean r^2^	Mean dist. (kb)	Max dist. (kb)	Chr	N pairs	Mean r^2^	Mean dist. (kb)	Max dist. (kb)
1	58,073	0.263	4.74	117.87	15	17,068	0.264	4.74	93.00
2	52,391	0.275	4.75	152.46	16	14,974	0.249	4.78	74.52
3	46,858	0.276	4.78	146.79	17	15,050	0.247	4.80	115.76
4	24,928	0.267	4.78	204.62	18	14,599	0.263	4.69	138.63
5	22,793	0.263	4.73	305.58	19	13,094	0.260	4.60	96.23
6	24,338	0.262	4.80	70.15	20	11,033	0.255	4.62	132.22
7	21,261	0.264	4.71	268.22	21	10,422	0.246	4.80	173.10
8	19,070	0.260	4.75	131.01	22	10,779	0.254	4.71	108.88
9	19,831	0.259	4.77	85.59	23	12,949	0.245	4.81	45.27
10	17,848	0.267	4.84	357.01	24	9,190	0.262	4.57	70.25
11	13,820	0.271	4.50	139.12	25	9,786	0.249	4.63	104.82
12	17,047	0.257	4.64	61.26	26	9,411	0.244	4.68	44.36
13	17,639	0.282	4.71	343.36	**All**	**507,918**	**0.263**	**4.74**	**357.01**
14	13,624	0.261	4.60	140.07					

*Chr* chromosome, *N pairs* number of SNP pairs, *Max dist.* maximum distance

### Effective population size

**Fig. 5:**
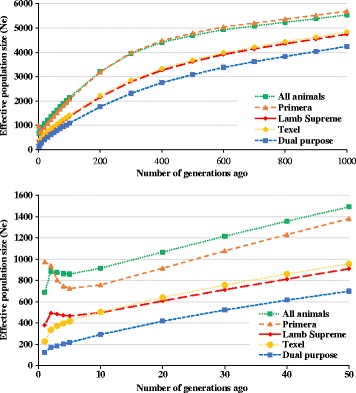
**a** Ancestral and recent effective population size in different time points in the past (Number of generations ago). **b** Ancestral and recent effective population size in different time points in the past (until 50 generations ago)

The N_e_ was evaluated for all animals together (*n* = 14,845) and separately by breed-group (Primera: *n* = 9,586; Lamb Supreme: *n* = 2,555; Texel: *n* = 1,661 and Dual-Purpose: *n* = 1,043) from the most recent generation to 1,000 generations ago (Fig. 5a, b and Additional file 3). The N_e_ ranged from 5,537 animals 1,000 generations ago to 687 in the most recent generation. The most recent N_e_ for all animals (687) and separately per breed-group: Primera (974), Lamb Supreme (380), Texel (227) and Dual-Purpose (125) was quite variable. For all breed-groups, N_e_ decreased over time, except for Primera and Lamb Supreme breed-groups, which increased over the last five generations.

### Consistency of gametic phase

**Fig. 6:**
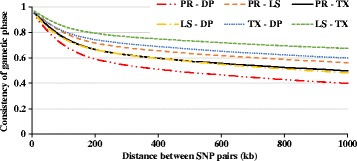
Consistency of gametic phase (Pearson correlations of signed r-values) at given distances for six selected breed-group pairs. PR: Primera, LS: Lamb Supreme, TX: Texel and DP: Maternal/Dual-Purpose

As presented in Fig. 6, the consistency of gametic phase was reasonably high among all breed-group pairs. Lamb Supreme and Texel presented the highest consistency of gametic phase. The lowest consistency of gametic phase was between Primera and Dual-Purpose breed-groups. At the SNP chip average distance between SNP, the consistency of gametic phase was higher than 0.94 for all breed pairs. At an average distance of 50 kb between SNP, the consistency of gametic phase between breed pairs was 0.81, 0.88, 0.85, 0.84, 0.87 and 0.90, for Primera – Dual-Purpose, Primera – Lamb Supreme, Primera – Texel, Lamb Supreme – Dual-Purpose, Texel – Dual-Purpose and Lamb Supreme – Texel, respectively.

### Principal component analysis

**Fig. 7:**
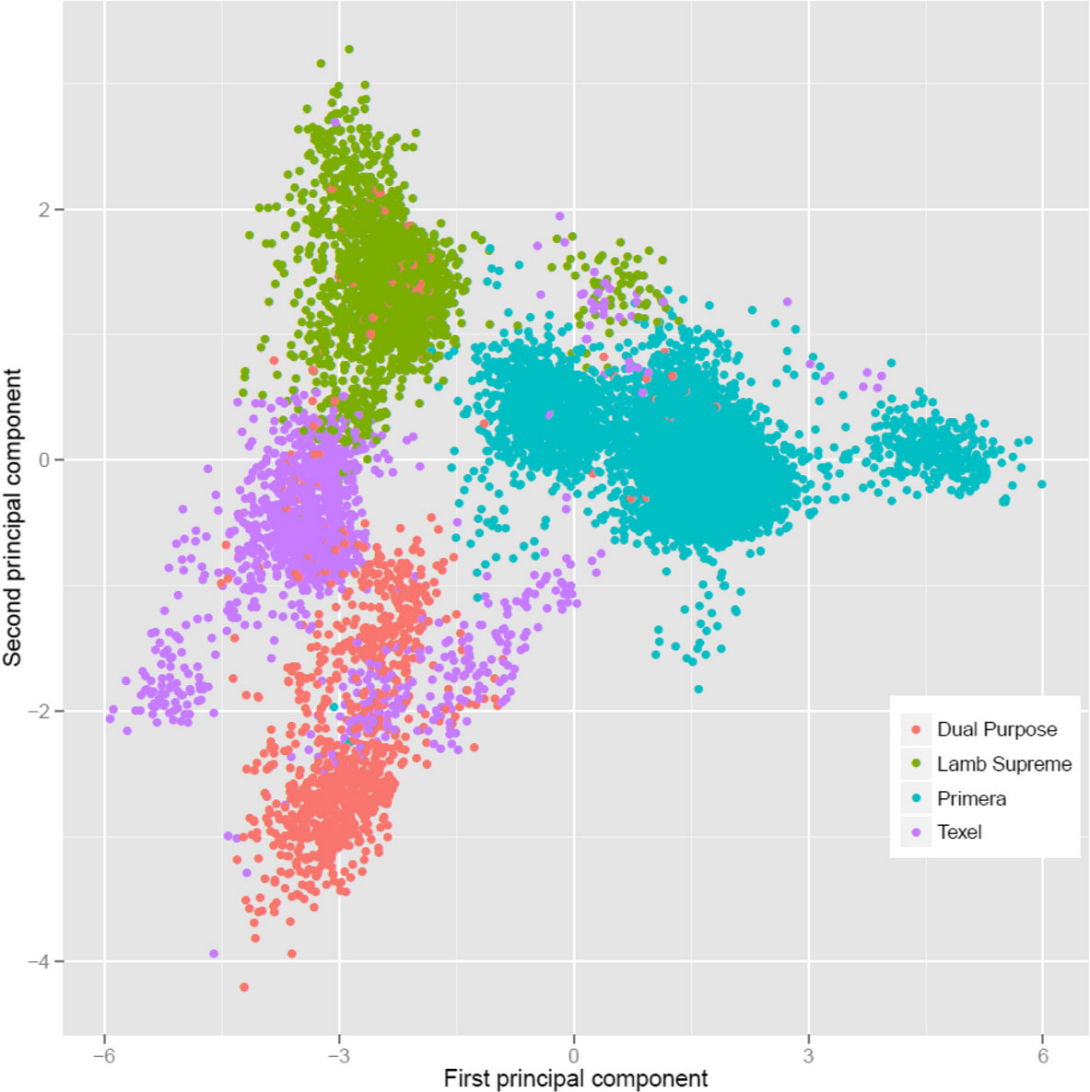
Principal component decomposition of the genomic relationship matrix colored by breed-group

To further understand the genetic relationships between single individuals and between breed-groups, we performed a principal component analysis (PCA) on the **G** matrix (Fig. 7). The plot of first and second principal components (PCs) did not show a clear discrimination between the breed-groups and an overlap among individuals from different breed-groups. The first and second PCs explained 5.14 and 4.91% of the total variance, respectively.

## Discussion

The short distance between adjacent SNPs is an advantage of the HD compared to lower density SNP chips, as in theory the markers would be closer to the QTL for the traits of interest and potentially in higher LD, allowing the markers to capture the QTL/causal mutations effects better and consequently increase the accuracies of mBVs predictions across breeds. The moderate MAF levels demonstrate the great genetic diversity of this population. However, these values can even be underestimated, because in the development of the HD SNP chip, a proportion of SNP with low MAF were included [6]. From the 517,902 SNPs that passed quality control, 82,859 (16%) of the SNPs had MAF less than or equal to 0.10. As shown in Fig. 3, the MAF ranges per breed group and across MAF bins were similar, indicating that ascertainment bias was likely small in these analyses [20].

Heterozygosity measures the level of genetic variation within a population with higher values indicating greater genetic variability. The mean H_e_ was high, revealing the great genetic diversity of this population. Similar estimates were reported by Beynon et al. [21] studying 18 Welsh breeds (average: 0.349). Al-Mamum et al. [22] reported levels of heterozygosity in Australian sheep breeds and crossbreds ranging from 0.30 to 0.40. Our results are also consistent with those reported by Kijas et al. [23] in a variety of world sheep breeds, with an average (± SD) of 0.33 (±0.03) and ranging from 0.22 (MacarthurMerino breed) to 0.38 (Rasa Aragonesa and Gulf Coast Native breeds). The high genetic diversity in this population can be explained by their breeding history. As described before, most of the composites were developed as non-breed specific composites and consequently, there was a big range of breeds involved in their formation. The haplotype sharing among the breeds contribute to the high genetic diversity observed in this study. Moreover, most of the genotyped animals are crossbred progeny from the composite breeds, which contribute to the increase in the genetic diversity seen.

Another aspect of interest while studying a commercial population under selection pressure is to study the level of inbreeding. The inbreeding coefficient of an individual is the probability that, at a given locus, an individual has received the same ancestral-allele from both parents [24]. It is known that genetic selection tends to increase inbreeding within a population [25] explicitly avoided in the mating decisions. The genotyped animals (*n* = 14,845) were outbred or had a low level of inbreeding on average (depending on the measure of inbreeding). However, there was a big range, indicating that there are inbred animals and this should be taken into account when planning matings in order to avoid high levels of inbreeding in the progeny. This can be implemented using a mating planning software to optimize the genetic contribution of each individual and control inbreeding at a target level.

**Fig. 8:**
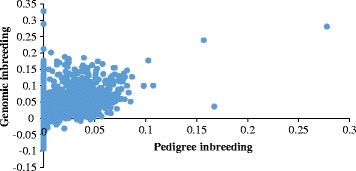
Genomic (F_VR_) and pedigree inbreeding coefficients for all the genotyped animals

As expected, some outbreeding (low inbreeding coefficients) was observed when estimating genomic inbreeding coefficients. The negative values correspond to animals with lower homozygosity than expected from the population MAFs. The low levels of inbreeding can be attributed to the high gene flow between different flocks by using outside sires (mainly Primera and Lamb Supreme flocks), recent composite breed formation, crossbreeding and reduced overlapping of generations. The majority of animals in this population are progeny from Primera and Lamb Supreme rams (Primera = 9,586, Lamb Supreme = 2,555, Texel = 1,661 and Dual-Purpose = 1,043). Both composites were recently developed based on a screening of a large number of animals from various flocks regardless of breed, which means that several breeds (and unrelated animals, consequently) contributed to the formation of these composites. Even though there was not a clear trend of increased inbreeding levels over years, it is important to continue monitoring this parameter. Genomic data could actually be used as an important tool to establish the genetic difference among rams in order to plan mating. As shown in Fig. 8, there were animals with pedigree inbreeding values of zero. However, their genomic level of inbreeding was much higher. The main reason for that is the pedigree incompleteness. Inbreeding levels should be taken into account when planning the matings in order to avoid inbreeding depression, as highlighted in several studies (e.g. [26, 27]).

### Extent of linkage disequilibrium

The levels of LD influences the power of QTL detection and accuracy of genomic predictions [4]. LD levels indicate the minimum number of markers for successful genomic predictions. Meuwissen et al. [3] in a simulation to predict genomic breeding values from dense markers across the whole genome with accuracies up to 0.85, found a required r^2^ level of 0.2. At the average marker spacing in the HD SNP chip (~5 kb) the average pairwise LD (r^2^) was 0.24. The results observed in this composite population indicate that genomic selection can be successfully implemented.

There is little knowledge about the degree of genome-wide LD in the sheep breeds included in this investigation. In a LD study including a collection of 74 sheep breeds and 49,034 SNP, Kijas et al. [23] observed a high variation in LD levels among breeds, with a Scottish breed (Soay) presenting the highest levels of LD and Qezel sheep (sampled in Iran) the lowest levels of LD. Using the HD SNP chip, Kijas et al. [6] reported LD levels at 10 kb of 0.186, 0.191, 0.279, 0.221 and 0.339 for Merino ewes, Merino sires, Poll Dorset, Suffolk and Border Leicester, respectively. For the population investigated in this study the LD levels at 10 kb were 0.179, smaller than estimates by Kijas et al. [6]. This is probably due to the high level of crossbreeding in this population and the wide genetic base used in the formation of the composites breeds.

The MAF distribution of the SNP influences estimates of LD [28]. Between pairs of low MAF loci, r^2^ tend to underestimate LD [29]. As mentioned by Kijas et al. [6], the SNPs chosen to be on the HD SNP chip were selected to have reasonable MAF and could introduce what is called ascertainment bias. This could affect the estimates of LD and N_e_. However, the authors evaluated the effect of low-frequency loci (MAF < 0.1) and observed that the removal of these SNPs caused a small inflation of r^2^ estimates. There are studies in dairy cattle showing that ascertainment bias in the estimation of LD using half-sib data might occur [30]. One alternative reported in dairy cattle is to use only maternal haplotypes for the LD and genetic diversity analysis [31]. However, in dairy cattle a single bull can have up to a million daughters due to the wide uptake of artificial insemination and half-sib families in genotype data are usually much larger compared to sheep datasets. In the present study, the average (range) number of progeny per sire was 17 (1–114) and there was a large number of sires (*n* = 877), which represented well the populations. To investigate potential overestimation of LD estimates, we also performed the analysis using a balanced dataset (removing extra progeny data per sire), in which the average (range) number of progeny per sire was 12 (1 – 17) and the total number of genotypes was reduced from 14,845 to 10,300 animals. The estimates from both analysis were statistically equal (*P* > 0.05), and therefore, only the results using the full dataset were presented.

The low levels of LD observed in the population investigated could be due to the fact that sheep domestication is likely to have involved a genetically broad sampling of their wild ancestors, and subsequent bottlenecks associated with breed formation were less severe than in other species as noted by Kijas et al. [23]. The low level of LD indicates a low level of selection intensity over generations. As reported in Fig. 4, the LD levels decrease as the distance between markers increased. However, it was noted two increases in LD levels (“bumps”) at short distances, which occurred around 2,400 and 2,700 generations ago. They could be associated with the process of domestication of the species. The archaeological evidences suggest that sheep were probably first domesticated approximately 8,000 – 9,000 years ago [32].

Even though there is a variation in LD levels per chromosome, the differences were small. The reason for that may be because most traits where an intense selection pressure was applied were polygenic traits and the breeding programs are still recent [33]. Differences in LD measures between chromosomes have been reported in other studies [34, 35]. These can be attributed to recombination rates varying between and within chromosomes, heterozygosity, genetic drift and effects of selection [34]. The differences between LD for each breed-group are consistent with their recent and past history of selection, as some breeds have smaller effective population size and consequently higher LD levels.

The low levels of LD observed in this study have practical applications for the implementation of genomic selection. It highlights the need to use a HD SNP chip for genomic predictions in a multi-breed population as the level of LD is relatively small even at short distances. A low-density panel could not capture enough LD to successfully predict mBVs in a multi-breed population as the one under investigation. Our results support the need for a HD SNP chip (i.e. 600 K) for genomic selection in this population. An alternative to reduce genotyping costs is to genotype lambs with low-density and impute to HD SNP chip, which has already been proven to be feasible in New Zealand multi-breed sheep populations [36].

#### Consistency of gametic phase

The improvement in accuracy of mBVs for a specific breed based on using data from other breeds (or breed-groups/crossbreds) depends on the consistency of gametic phase between the SNP and QTL across breeds and on the similarity of QTL effects between breeds. The more distant the relationship between individuals, the shorter the genomic distance over which the phase will be consistent. As presented in Fig. 6, the consistency of gametic phase was reasonably high among all breed-group pairs. Lamb Supreme and Texel presented the higher consistency of gametic phase, which was expected as Lamb Supreme also included Texel haplotypes in its formation (as described in the “Genetic Resources” section, Additional file 1). The lowest consistency of gametic phase was between Primera and Dual-Purpose breed-groups, which is consistent with the Primera breed development history. The Primera composite breed did not include animals from Dual-Purpose breeds in its formation, compared to the Lamb Supreme which included animals from Romney and Coopworth blood lines, consequently the genetic relationship between Primera and Dual-Purpose was expected to be lower. However, the still moderate to high levels of consistency of gametic phase is due to that most Terminal sires were mated to maternal/Dual-Purpose breeds, as part of progeny testing, therefore, the progeny (majority of genotyped animals) were genetically connected to some extent. These results suggest that better accuracies of genomic predictions could be attained when using a mixed training population as the SNP effects seem to be similar at some extent among breed-groups.

#### Principal component analysis

Principal Component Analysis were used to visualize and explore the genetic relationships among individuals and breed-groups. Basically, PCA absorbs the information of allele frequencies into a small number of synthetic variables, facilitating the interpretation of population structure. PCA analysis showed that most breed-groups formed overlapping clusters and they are not clearly separated populations. The genetic closeness between these animals is probably due to crossbreeding and exchange of genetic material (see Additional file 1).

#### Effective population size

Changes in the effective population size reflect past events that occurred in the corresponding populations. N_e_ provides an insight about the breeds’ evolution and is another relevant factor to the accuracy of genomic predictions of mBVs. A smaller N_e_ is associated with a higher LD level and expected accuracy of linkage disequilibrium [4]. The N_e_ is also an important parameter in predicting theoretical accuracies [37] and consequently to estimate the size of the training population required to achieve specific accuracies for future selection. There are no published estimates of N_e_ for the New Zealand Terminal Sire composites.

The N_e_ has decreased over time (Fig. 5), which is probably due to natural and artificial selection. The dramatic decrease in N_e_ in the most recent generations could be due to different reasons such as the variety of breeds used to develop New Zealand Composite breeds, the reduction in the size of the New Zealand population in the last 30 years and to an increase in selection intensity in the national breeding programs. However, there was an increase in N_e_ for the Primera breed-group in the most recent generations, which is probably due to the introduction of outside rams and a high level of crossbreeding (Additional file 1). The recent N_e_ for all animals (687) and separately per breed-groups: Primera (974), Lamb Supreme (380), Texel (227) and Dual-Purpose (125) was quite variable. The N_e_ observed for this population is quite high indicating the genetic variability of this population. Kijas et al. [23] reported a N_e_ estimate for New Zealand Texel of 282. For the other composite breeds, we are reporting N_e_ estimates for the first time. However, Table 3 presents the main breeds (and their N_e_ based on literature estimates) involved in the formation of the composites Primera, Lamb Supreme and Dual-Purpose.

**Table 3:** Effective population size (N_e_) for composite breeds and N_e_ for their ancestor breeds reported in the literature

Composite breed (N_e_)	Ancestor breeds	N_e_
Lamb Supreme (380)	Poll-Dorset	318^a^
	Wiltshire	100^a^
	Romney	405^a^
	Dorset	134^a^
	Coopworth	98^b^
	Texel	282^a^
Primera (974)	Suffolk	569^a^
	Poll-Dorset	318^a^
	Dorper	264^a^
	Hampshire	-
	Dorset	134^a^
Dual-Purpose (125)	Texel	282^a^
	Lamb Supreme	380^c^
	Romney	405^a^
	Perendale	109^b^
	Finn	795^a^
	Coopworth	98^b^
	Poll-Dorset	318^a^
	East Friesian	186^a^

^a^Kijas et al. [23]; ^b^Vincent Prieur, AgroParisTech and AgResearch, Master dissertation; ^c^current study

Kijas et al. [23] reported recent N_e_ for several sheep breeds from 100 (Wiltshire breed) to 1,317 (Qezel breed). The authors revealed that 25 breeds have N_e_ exceeding 500 and only two showed evidence of a narrow genetic base (N_e_ < 150), which is consistent with our findings. In general, sheep breeds have a higher level of genetic diversity compared to other species such as dairy cattle (e.g. N_e_ for Holstein = 99), suggesting a highly diverse population prior to domestication and that genetic bottlenecks were not as intensive as in other species [38].

The high genetic diversity and effective population size observed in this population implies that selection response for growth, carcass and meat quality traits may be expected to continue in the long term and higher genetic responses may be achieved compared to more homogeneous populations. Goddard and Hayes [39] showed that more animals are needed for training to obtain the same accuracy with increasing N_e_. Therefore, the N_e_ estimates observed in this study also has implications for genomic selection, as genetic diversity is a key indicator of the required size of training population that is needed to achieve accurate genomic predictions. To ensure an animal population is long-term viable, a threshold of N_e_ = 100 has been given [40]. Our results of current effective population size are above the threshold, indicating the great genetic diversity of this population.

## Conclusions

This study reports, for the first time, estimates of linkage disequilibrium, genetic diversity, and population structure parameters from a genome-wide perspective in New Zealand Terminal Sire composite sheep breeds. Even though high genetic diversity was observed in this population, the observed levels of LD indicate that genomic selection could still be successfully implemented. The moderate to high consistency of gametic phase between breed-groups support the pooling of the animals in a mixed training population for genomic predictions. Effective population size seems to have been decreasing over time, however it is still high, highlighting the need for genotypes and phenotypes from a large number of animals in order to capture the haplotype diversity and increase accuracies of genomic predictions. Even though the average inbreeding levels were low, it is important to consider this information when planning matings, as there are some highly inbred animals. The results reported herein are a first step toward understanding the genomic architecture of a Terminal Sire composite sheep population and for the optimal implementation of genomic selection and genome-wide association studies in these sheep populations.

## Additional files

Additional file 1: Genetic resources: composite breeds’ history. (DOCX 19 kb)
Additional file 2: Average linkage disequilibrium (r^2^) between adjacent SNP pairs by chromosome and per each sire breed-group. (DOCX 14 kb)
Additional file 3: Ancestral and recent effective population size. (DOCX 17 kb)

## Abbreviations

CT: Computed tomography
DNA: Deoxyribonucleic acid
eBV: Estimated breeding value
G: Genomic relationship matrix
Gb: Giga base pairs
GS: Genomic selection
HD: High density
He: Heterozygosity
kb: Kilo base pairs
LD: Linkage disequilibrium
MAF: Minor Allele Frequency
Mb: Mega base pairs
mBV: Molecular breeding value
Ne: Effective population size
PCA: Principal component analysis
QTL: Quantitative trait loci
SD: Standard deviation
SNP: Single nucleotide polymorphism
