# Supplementary material for: Genetic diversity of a New Zealand multi-breed sheep population and composite breeds’ history revealed by a high-density SNP chip
Source: BMC Genet. 2017 Mar 14;18:25. doi: 10.1186/s12863-017-0492-8 (PMC5348757; doi:10.1186/s12863-017-0492-8)
Supplement: Additional file 1: — Genetic resources: composite breeds’ history. (DOCX 19 kb) [file 12863_2017_492_MOESM1_ESM.docx]

***Genetic resources***

In this section we present a brief history of the Primera, Lamb Supreme, Landmark and Highlander composites will be presented ([1-3] and personal communication: Natalie Pickering and John McEwan), due to the lack of literature for these breeds.

**Lamb Supreme:** The Lamb Supreme breeding programme was the industry’s first white-faced, non-breed specific terminal sire composite developed in New Zealand. It was established in 1989 and 1990 with the screening 0.8% of over 500,000 high live weight (measured October to December) ewe hoggets (regardless of breed) from Landcorp’s commercial farms throughout New Zealand. Over 3 years (1990-1992) a range of industry-bred white-faced rams selected for objectively-measured live weights and carcass traits were used. Breeds included lean-selected Poll Dorset, Wiltshire, Romney x Dorset, Coopworth, Texel and high-growth Romney. The resulting crossbred progeny were named as “Lamb Supreme”. The relevance given to the white-face breeds was due to the fact that they could cross-over and be used as an input into maternal/dual purpose breeds. Computed tomography (CT) scanning started in 1995 in both the Landcorp Texel flocks and Lamb Supreme programmes, and these were the only breeding programmes in New Zealand that have routinely used this procedure since that time. The top 10 - 15% of ram lambs were CT scanned to measure meat yields in the primal regions (loin, hind leg and shoulder) and all lambs were also measured for ultrasonic eye muscle traits at 4 to 6 months of age. This new terminal sire composite has been used widely throughout New Zealand. Lamb Supreme and Texel have been involved in FarmIQ Progeny Testing and genomic selection for various growth, carcass and meat quality traits is under implementation.

**Primera:** The Primera composite was also a non-breed specific terminal sire breed. Breeds that have contributed to the formation of Primera include: Suffolk, Poll Dorset, Australian White Suffolk, Dorper, Hampshire and Dorset Down. The Primera is a coloured-face composite breed and used progeny testing based selection. The coloring means a wider range of terminal sire breeds could be accessed and commercial farmers appreciate its ability to mark non-breeding stock. This composite breed had been objectively selected for growth and carcass characteristics of their progeny. The main focus was on traits that are related to higher saleable meat yields which maintain sufficient fat covers and produce individual cuts with desirable conformation. The Primera genetic improvement programme was unique, as estimated breeding values (eBVs) for all carcass traits were based on progeny performance, not estimated from sires’ own performance, making them highly accurate eBVs. Before rams could be used in the programme they were progeny tested across a maternal ewe base. Progeny Testing started in 2002 and up to 120 ram lambs were progeny tested at its peak. Since the 2011 progeny test, the measurements were reduced to only include butt circumference, fat depth at 12^th^ rib, and collection of loins for meat quality measurements (under the FarmIQ programme). Before 2012, progeny test data was sent to AgResearch (Mosgiel, New Zealand) for calculation of eBVs for carcass weight, eye muscle area, primal cut weight (tenderloin + boneless loin weight) and fat depth at 12^th^ rib. These eBVs were used in the selection index, plus an eBV for birth weight was considered on the side in the final sire selections. Since December 2012, the selection index for the Primera changed to the “Focus Genetics Terminal index” and the best ~50 ram lambs from the elite flocks were CT scanned on top of ultra-sound scanning of eye muscle area already undertaken. The Primera terminal sire composite has been widely used in New Zealand and exported to other countries such as United Kingdom and Australia.

**Maternal/dual purpose breeds:** the most common maternal/dual purpose breeds that contributed to this population were Coopworth, Romney, Highlander and Landmark. For convenience, here after, maternal/dual purpose breeds will be referred just as dual purpose. A description of the composite breeds Landmark and Highlander is:

**Landmark:** The Landmark programme was established to develop a higher-performing (in fertility, and growth particularly) maternal composite. It began in 1998/99 by screening in 12% of 55,000 commercial Landcorp ewe lambs sired by Texel or Lamb Supreme rams (out of predominantly Romney and some Perendale ewes) based on weaning weight (accounting for birth rank where possible).

A total of 74 industry-sourced rams comprising 10 maternal breeds and crosses were used over these young ewes for about 4 seasons. Ram breeds included Finnish x Coopworth, Finnish x Poll Dorset, East Friesian x Coopworth, East Friesian x Poll Dorset, and straight Coopworth and Poll Dorset rams. Preference was given to rams sourced from recorded flocks. The resulting crossbred ram lambs from these matings were fully recorded and selected for use over Romney ewes representatively sampled out of the Waihora Romney breeding programme [1]. The progeny from these matings were classed as Landmarks. The breed makeup of the Landmark was presumed to be about ~60% Romney, ~15% Lamb Supreme, ~15% Texel, with the remaining 10% coming from combinations of Coopworth, Dorset, Finnish and Friesian (i.e., about 2-3% of each of these breeds).

**Highlander:** The Highlander is a highly fertile maternal composite sheep that had been objectively selected for high reproductive performance, growth and carcass characteristics. The breed composition of the Highlander is made up of ¼ Romney, ¼ Texel and ½ Finnish Landrace. It was started by Andy Ramsden in the early 1990s who initially put Finnish across his Romney ewes and eventually added Texel to the mix. The Highlander is a no-flush ewe, through selecting for number of lambs born and historically culling all ewes that conceived in the second cycle. Both maternal composites (Highlander and Landmark) are now using a breeding objective for reproduction and fertility including hoggets survival, growth fleece weight and also resistance to facial eczema in the Highlander composite.

**References**

1. Nicoll G: **Performance and financial returns of two Romney flocks sired by Waihora or Commercial rams**. *New Zealand Journal of Agricultural Research* 1989, **32**(1):37-43.

2. Nicoll G: **Living Legend Address: The sire-breeding programmes of the Department of Lands and Survey and Landcorp Farming Limited**. In: *Proceedings of the New Zealand Society of Animal Production 2014 2014; Napier, New Zealand*. 124-132.

3. Nicoll G, Alderton M, Annandale D, Coleman I, Grimwood T, Thomson J: **A terminal sire breeding programme based on screening for hogget liveweight**. In: *Proceedings of the New Zealand Society of Animal Production: 1992*. 125-127.
