# Supplementary material for: Genetic diversity of a New Zealand multi-breed sheep population and composite breeds’ history revealed by a high-density SNP chip
Source: BMC Genet. 2017 Mar 14;18:25. doi: 10.1186/s12863-017-0492-8 (PMC5348757; doi:10.1186/s12863-017-0492-8)
Supplement: Additional file 2: — Average linkage disequilibrium (r2) between adjacent SNP pairs by chromosome and per each sire breed-group. (DOCX 14 kb) [file 12863_2017_492_MOESM2_ESM.docx]

| **Table S1.** Average linkage disequilibrium (r^2^) between adjacent SNP pairs by chromosome and per each sire breed group. | | | | | | |
| --- | --- | --- | --- | --- | --- | --- |
|  | **Sire breed group** | | | |  | **All animals** |
| **Chromosome** | **Lamb Supreme** | **Primera** | **Texel** | **Dual purpose** |  |  |
| **1** | 0.266 | 0.255 | 0.262 | 0.275 |  | 0.263 |
| **2** | 0.274 | 0.268 | 0.266 | 0.286 |  | 0.275 |
| **3** | 0.278 | 0.268 | 0.275 | 0.288 |  | 0.276 |
| **4** | 0.270 | 0.260 | 0.268 | 0.279 |  | 0.267 |
| **5** | 0.265 | 0.256 | 0.263 | 0.273 |  | 0.263 |
| **6** | 0.265 | 0.254 | 0.260 | 0.274 |  | 0.262 |
| **7** | 0.267 | 0.256 | 0.261 | 0.276 |  | 0.264 |
| **8** | 0.263 | 0.254 | 0.258 | 0.271 |  | 0.260 |
| **9** | 0.263 | 0.252 | 0.259 | 0.274 |  | 0.259 |
| **10** | 0.267 | 0.261 | 0.265 | 0.279 |  | 0.267 |
| **11** | 0.274 | 0.264 | 0.265 | 0.280 |  | 0.271 |
| **12** | 0.259 | 0.251 | 0.256 | 0.267 |  | 0.257 |
| **13** | 0.289 | 0.274 | 0.278 | 0.295 |  | 0.282 |
| **14** | 0.265 | 0.254 | 0.258 | 0.270 |  | 0.261 |
| **15** | 0.267 | 0.257 | 0.260 | 0.278 |  | 0.264 |
| **16** | 0.253 | 0.242 | 0.247 | 0.259 |  | 0.249 |
| **17** | 0.250 | 0.241 | 0.243 | 0.254 |  | 0.247 |
| **18** | 0.267 | 0.256 | 0.261 | 0.275 |  | 0.263 |
| **19** | 0.260 | 0.253 | 0.258 | 0.268 |  | 0.260 |
| **20** | 0.262 | 0.248 | 0.257 | 0.266 |  | 0.255 |
| **21** | 0.251 | 0.240 | 0.244 | 0.252 |  | 0.246 |
| **22** | 0.257 | 0.247 | 0.253 | 0.263 |  | 0.254 |
| **23** | 0.251 | 0.237 | 0.243 | 0.258 |  | 0.245 |
| **24** | 0.268 | 0.254 | 0.261 | 0.276 |  | 0.262 |
| **25** | 0.254 | 0.242 | 0.249 | 0.255 |  | 0.249 |
| **26** | 0.250 | 0.236 | 0.242 | 0.256 |  | 0.244 |
| **All** | **0.266** | **0.256** | **0.261** | **0.274** |  | **0.263** |
