# Supplementary material for: Genetic diversity of a New Zealand multi-breed sheep population and composite breeds’ history revealed by a high-density SNP chip
Source: BMC Genet. 2017 Mar 14;18:25. doi: 10.1186/s12863-017-0492-8 (PMC5348757; doi:10.1186/s12863-017-0492-8)
Supplement: Additional file 3: — Ancestral and recent effective population size. (DOCX 17 kb) [file 12863_2017_492_MOESM3_ESM.docx]

| **Table S1.** Ancestral and recent effective population size. | | | | | |
| --- | --- | --- | --- | --- | --- |
| **Generations ago** | **All animals** | **Primera** | **Lamb Supreme** | **Texel** | **Dual purpose** |
| 1,000 | 5,537 | 5,684 | 4,742 | 4,841 | 4,245 |
| 900 | 5,377 | 5,520 | 4,543 | 4,627 | 4,030 |
| 800 | 5,234 | 5,362 | 4,353 | 4,439 | 3,830 |
| 700 | 5,081 | 5,198 | 4,141 | 4,213 | 3,616 |
| 600 | 4,931 | 5,041 | 3,913 | 3,985 | 3,381 |
| 500 | 4,693 | 4,786 | 3,611 | 3,684 | 3,081 |
| 400 | 4,404 | 4,474 | 3,261 | 3,328 | 2,754 |
| 300 | 3,948 | 3,983 | 2,798 | 2,853 | 2,312 |
| 200 | 3,204 | 3,192 | 2,160 | 2,221 | 1,765 |
| 100 | 2,142 | 2,072 | 1,368 | 1,418 | 1,090 |
| 90 | 2,016 | 1,939 | 1,280 | 1,328 | 1,016 |
| 80 | 1,889 | 1,802 | 1,190 | 1,241 | 940 |
| 70 | 1,758 | 1,663 | 1,096 | 1,147 | 862 |
| 60 | 1,628 | 1,525 | 1,004 | 1,055 | 782 |
| 50 | 1,493 | 1,380 | 910 | 956 | 700 |
| 40 | 1,357 | 1,231 | 813 | 861 | 615 |
| 30 | 1,217 | 1,078 | 714 | 757 | 523 |
| 20 | 1,067 | 916 | 608 | 641 | 418 |
| 10 | 916 | 759 | 499 | 504 | 292 |
| 5 | 860 | 725 | 467 | 417 | 218 |
| 1 | 687 | 974 | 380 | 228 | 125 |
